# Supplementary material for: Comparative transcriptomics reveal differential gene expression among Plasmodium vivax geographical isolates and implications on erythrocyte invasion mechanisms
Source: PLoS Negl Trop Dis. 2024 Jan 29;18(1):e0011926. doi: 10.1371/journal.pntd.0011926 (PMC10901308; doi:10.1371/journal.pntd.0011926)
Supplement: Attachment — Submitted filename: Responses to Reviewers.pdf [file pntd.0011926.s003.pdf]

<u>Reviewer comments in italics and our responses in bold:</u>

1. Comparing Plasmodium vivax transcriptomes obtained with different study protocols is necessarily challenging. This author might perhaps discuss the potential limitations of the comparative approach in greater detail.

Our response: **We have added this technical limitation to the discussion (lines 423-426).**

2. In two of the studies (Ethiopia and Brazil), cryopreserved, patient-derived isolates were thawed and maintained in short-term culture ex-vivo for approximately 40-44 hours, under slightly different conditions, for schizont maturation; in one study (Cambodia), fresh parasites were used for ex-vivo maturation. The key question is whether differences in transcriptomic profiles among parasites were mostly due to the geographic origin of parasites (Horn of Africa vs. Southeast Asia vs. Amazon Basin in South America) or to differences in sample preparation protocols prior to transcriptomic analysis. This question deserves deeper discussion throughout the manuscript.

Our response: **We thank the reviewer for highlighting an unanswered question in our manuscript. We emphasized in our materials and methods that we processed these samples using identical bioinformatics pipelines to avoid as much bias as possible and ensured schizont proportions were not significantly different (lines 221-226). Currently, there is not a study examining expressional disruptions between cryopreserved and fresh *ex-vivo* maturation. We have emphasized this technical limitation and knowledge gap, while showing a desire to explore this unanswered question (lines 423-429).**

3. Interestingly, CIBERSORTx deconvolution analysis shows the presence of some ring-stage transcripts in Brazilian isolates (Figure 1), which is unexpected after the ex-vivo maturation process. However, the difference in the proportion of late trophozoites and schizonts among samples of different geographic origin did not reach statistical significance.

Our response: **We have mentioned this in the discussion as our limitations (lines 425-426).**

4. Why are transcriptomes of Cambodian and African parasites more similar to each other than they are to those from South American samples is not entirely clear. Population genomic analyses (e.g., doi: 10.1038/ng.3588) typically show greater similarity between African and American lineages of P. vivax , compared with Southeast Asian lineages. Again, can this be due to differences in study protocols used for samples processing and transcriptomic analyses rather than geographic differences in transcription patterns?

Our response: **We have attempted to explain the observed transcriptional pattern between geographical isolates in our discussion. Although it remains largely**

unclear, we offered several explanations including genetic relatedness among geographical isolates and technical processing of samples (lines 403-429).

5. Because the genes coding for the PIR family in P. vivax had originally been described as the vir multigene family in P. vivax, the nomenclature may be a bit confusing.

Our response: We have clarified the nomenclature to avoid potential confusion (line 77).

6. The authors correctly state that "malnourishment has a protective effect to P. vivax infections in people from the western Brazilian Amazon (70)". Yes, there is a single cross-sectional report of such an association in children, with suboptimal controlling for confounders, which cannot be taken as evidence for a causal association between malnutrition and protection from malaria. I suggest these data are not overinterpreted in this context (differences between the transcriptomes of two P. vivax lineages from adults in Brazil and those from other geographic regions) as population-based nutritional status data from the study sites are not considered here.

Our response: We thank the reviewer for their insightful comment. To avoid potentially incorrect correlations, we have removed this as an explanation of genetic profiles but emphasized the need to further explore malnutrition as a causative agent (lines 419-421).
